# Intraocular pressure *vs* intracranial pressure in disease conditions: A prospective cohort study (Beijing iCOP study)

**DOI:** 10.1186/1471-2377-12-66

**Published:** 2012-08-03

**Authors:** Zhen Li, Yingxin Yang, Yan Lu, Dachuan Liu, Erhe Xu, Jianping Jia, Diya Yang, Xiaojun Zhang, Huiqing Yang, Daqing Ma, Ningli Wang

**Affiliations:** 1 Beijing Tongren Eye Center, Beijing Tongren Hospital, Capital Medical University, Beijing, 100730, China; 2 Department of Ophthalmology, Xuan Wu Hospital, Capital Medical University, Beijing, 100053, China; 3 Guang’anmen Hospital, China Academy of Chinese Medical Sciences, Beijing, 100053, China; 4 Department of Neurology, Xuan Wu Hospital, Capital Medical University, Beijing, 100053, China; 5 Department of Neurology, Beijing Tongren Hospital, Capital Medical University, Beijing, 100730, China; 6 Anaesthetics, Pain medicine and Intensive Care, Imperial College London, Chelsea and Westminster Hospital, London, SW10 9NH, UK

**Keywords:** Intracranial pressure, Intraocular pressure, Lumber puncture, Tonometer

## Abstract

**Background:**

The correlation between intracranial pressure (ICP) and intraocular pressure (IOP) is still controversial in literature and hence whether IOP can be used as a non-invasive surrogate of ICP remains unknown. The aim of the current study was to further clarify the potential correlation between ICP and IOP.

**Methods:**

The IOP measured with Goldmann applanation tonometer was carried out on 130 patients whose ICP was determined via lumber puncture. The Pearson correlation coefficient between ICP and IOP was calculated, the fisher line discriminated analysis to evaluate the effectivity of using IOP to predict the ICP level.

**Results:**

A significant correlation between ICP and IOP was found. ICP was correlated significantly with IOP of the right eyes (p < 0.001) and IOP of the left eyes (p = 0.001) and mean IOP of both eyes (p < 0.001), respectively. However, using IOP as a measurement to predict ICP, the accuracy rate was found to be 65.4%.

**Conclusion:**

Our data suggested that although a significant correlation exists between ICP and IOP, caution needs to be taken when using IOP readings by Goldmann applanation tonometer as a surrogate for direct cerebrospinal fluid pressure measurement of ICP.

## Background

Intracranial pressure (ICP) is an essential measurement in disease diagnosis for the central nervous system [1]. Elevated ICP is often the initial signal of some life threatening conditions such as traumatic brain injury, mass effect from tumors or various hemorrhagic catastrophes. Currently, direct measurement of cerebrospinal fluid pressure (CSFP) by lumbar puncture or catheter manometer of intraparenchymal or intraventricle pressure is considered to be the “gold standard” of ICP measurement. However, the direct measurement of ICP is not without risk due to its invasiveness and potential risk of intracranial hemorrhage, or even cerebral herniation and hence cannot be widely used as a matter of safety concerns. The noninvasive methods of ICP measurement such as transcranial Doppler (TCD), visual-evoked responses (VERs), brain stem auditory- evoked responses (BAERs), ophthalmodynamometry, scalp blood flow (SBF) measured by Laser Doppler, and impedance audiometry are still explored at pre-clinical stage or require further validation [2-6]. Moreover, the limitations of these examinations including special equipment requirements and time consumption may likely prevent adoption for a wide clinical use. Recent publications suggest a strong correlation between ICP and intraocular pressure (IOP) [7-9], which, however, is not supported by other previous studies [10-12]. In this study, we have mesured ICP and IOP on 130 patients to investigate whether the non-invasive IOP measurement could be an effective surrogate for direct CSFP measurements of ICP.

## Methods

This was a hospital-based prospective cohort study conducted at two university affiliated hospitals from May 2010 to May 2011. The Medical Ethics Committees of the Xuanwu Hospital and Beijing Tongren Hospital, Capital Medical University, Beijing, China, approved the study protocol. With their informed consent obtained, one hundred and seventy-five patients who underwent lumber puncture due to different neurological symptoms or neurological diagnoses were enrolled in this study.

Patients with glaucoma and history of intraocular operation or ocular disease that influenced intraocular pressure measurement were excluded from the study. The exclusion criteria also included history of intracranial surgery and spinal cord disease. Other reasons causing a mis-measurement of CSFP by lumber puncture, and medicine intake, such as mannitol, carbonic anhydrase inhibitors, β-blockers, that would influence either ICP or IOP were also excluded. According to this exclusion criteria, 2 patients with glaucoma, 1 patient with intraocular silicone oil persistence, 1 patient with severe pterygium, 13 patients with history of intraocular surgery for cataract treatment, 16 patients with history of intracranial operation, and 5 patients with occupying lesion in the spine diagnosed by MRI and 7 patients with medication administration were excluded. The remaining 130 patients were included in the final data analysis. The causes of lumber puncture are shown in Table1.

**Table 1:** The reasons for CSF pressure measurement viaa Lumber Puncture

**Cause of lumber puncture**	**n**	**%**
headach	49	37.7
Acroparesthesia	27	20.8
Limbs anergy	19	14.6
Meningitis review	7	5.4
Convulsion	4	3.1
Venous sinus thrombosis review	4	3.1
Locomotion disability	4	3.1
Diplopia	3	2.3
Language disorders	2	1.5
Loss of memory	2	1.5
limb’s muscle atrophy	2	1.5
facial palsy	2	1.5
mental disorder	1	0.8
bucking during drink	1	0.8
difficult to swallow	1	0.8
heteronomous shaking	1	0.8
paroxysmal neck turning to the side.	1	0.8
total	130	100

Before lumber puncture examination, patient’s IOP was measured 3 times with a Goldmann applanation tonometer under topical anesthesia and the mean value of three was used for data analysis. All patients were then followed with CSFP measurement. All lumber puncture and CSFP measurement with a standard method that was described in our previous work [13] were performed during 8 to10 AM.

The data was presented as Mean ± SD. The Pearson correlation coefficient for assessing the correlation between ICP and IOP and the fisher line discriminant analysis for evaluating the accuracy rate to predict ICP with IOP were performed with SPSS software (SPSS for Windows, version 13.0, SPSS, Chicago, IL, USA). A P value less than 0.05 was considered to be of statistical significance.

## Results

Of the remaining 130 patients, 74 cases (56.9%) were men and 56 cases (43.1%) were women. The patients’ epidemic data and measured IOP and ICP values are presented in Table2. Among them, 2.3% (3/130) of patients had decreased ICP less than 5 mmHg, 68.5% (89/130) of patients had normal ICP between 5 and 15 mmHg, and 29.2% (38/130) of patients with the elevated ICP more than 15 mmHg. Regarding MIOP values, 6.9% (9/130) of patients had lower IOP, less than 10 mmHg; 90.8% (118/130) of patients with normal IOP between 10 and 21 mmHg,the other 2.3% (3/130) of patients with higher IOP more than 21 mmHg.

**Table 2:** Patients’ epidemic data

	**minimum**	**maximum**	**mean ± SD**
Age (year)	12	26	37 ± 15
BMI (kg/m2)	16.9	37.1	23.8 ± 4.0
IOP-OD (mmHg)	8.1	27.0	14.40 ± 3.05
IOP-OS (mmHg)	8.3	22.5	14.40 ± 2.72
MIOP (mmHg)	8.4	24.8	14.40 ± 2.78
ICP (mmHg)	3.31	29.41	12.79 ± 5.34

**Figure 1:**
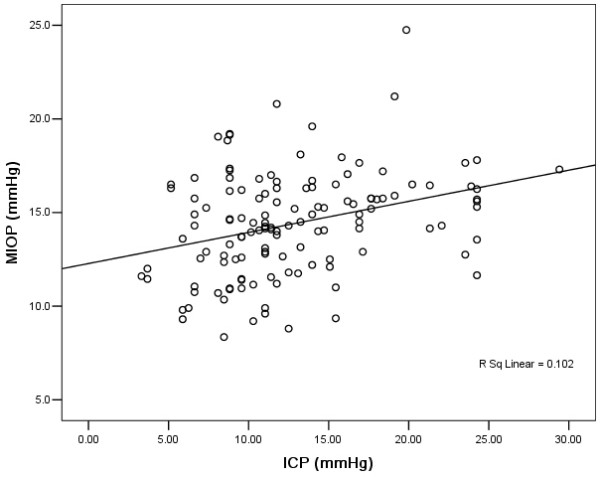
**The scatter plot of ICP (mmHg) measurement versus MIOP (mmHg) measurement with a positive correlation (r = 0.320, P <** **0.001).**

Our results showed that ICP was significantly and positively correlated with IOP. ICP was correlated significantly with IOP of the right eyes (r = 0.33, P < 0.001) and IOP of the left eyes (r = 0.29, p = 0.001) and mean IOP (MIOP) of both eyes (Figure1) (r = 0.320, p < 0.001), respectively. A significant correlation between ICP and BMI (r = 0.52, p < 0.001) but no correlation between ICP and age (p = 0.095, r = −0.147) were found.

When ICP of 200 mmH_2_0 (15 mmHg) was used as the cutoff point, the patients then were divided into two groups (ICP ≤ 15 mmHg vs > 15 mmHg) for further analysis to see whether IOP can predict the patients’ ICP (Table3).

**Table 3:** The results of using IOP measurement to evaluated the ICP level

***Primary group***	***Predicted group***		**total**
	**Group1**	**Group2**	
Group1	59	33	92
Group2	12	26	38
**total**	71	59	130

Using fisher line discriminant analysis, two discriminant equations were obtained:Group1:ICP = 1.926 × MIOP-14.113;Group2:ICP = 2.147 × MIOP-17.361. The cutoff point of discrimination is 0.5. The wilk’s lambda value of this function is 0.931 (p = 0.003) with 65.4% of original grouped cases that has been correctly classified.

## Discussion

We examined the relationship between the ICP measured by lumber puncture and the IOP determined by applanation tonometer on 130 patients and found a significant correlation between ICP and IOP (r = 0.32, p < 0.001) which is in line with previous published studies [13][14]. In a recent study with 76 concurrent ICP and IOP measurements in 27 patients in emergency medicine settings, all patients with an abnormal ICP had an abnormal IOP and *vice versa*[7] which was well supported by the work conducted in children with severe head injuries [9]. In Rhesus monkeys, experimentally induced raised ICP caused an increase in IOP [15]. ICP and IOP changed in parallel during induced respiratory acidosis and alkalosis was also well documented [16]. In an experiment on dogs, prostaglandin (E1, E2, A2) infusions induced a decrease of both ICP and IOP [17]. Taken together, all studies in animals and humans, one may conclude that IOP could predict ICP to some extent. There are also anatomical and physiological reasons for both pressures reflecting one to the other regardless of disease status: 1) The cerebrospinal fluid (CSF) in optic nerve subarachnoid space communicates with CSF in the brain at the site of the chiasmatic cistern. Direct pressure transmission through the CSF surrounding the optic nerve sheath exactly at the point where the optic nerve enters the orbit; 2) Most blood in optic veins backflow into the intracranial cavernous sinus through the superior orbital fissure. Increased ICP causes increased ophthalmic venous pressure, which would be transmitted directly to the ocular fluid, thus increasing the IOP [18]. In addition, A neuroimaging study showed a significant shorter axial length of the eye globes in patients with idiopathic intracranial hypertension compared with control subjects [19]. The enlarged retrobulbar optic nerve most probably exerted anteroposterior compression on the globe as evidenced by the posterior sclera flattening associated with intracranial hypertension [20]. It was the compressed eye globe volume that caused increased IOP. IOP and ICP have a similar physiological pressure range and similar response to changes in intraabdominal and intrathoracic pressure [21]. The fast changes in IOP and ICP by a probable alteration of intraocular and intracranial blood volumes was also reported [16].

However, we only found IOP in about two-thirds of the cases (65.4%) can correctly predict ICP level in this group of participants. The IOP and ICP are two interrelated and relatively independent pressure systems. IOP and ICP keep themselves in a relatively stable state through aqueous circulation and CSF circulation and their corresponding neural regulatory mechanisms respectively. Therefore, either a mild change in ICP cannot cause a considerable change in IOP which has been demonstrated clearly in a previous study [14] or there is even no correlation between IOP and ICP as reported previously [10]. In an elegant study using complicated measures to determine the relationship between invasive continuous monitoring of ICP using the intraparenchymal sensor and IOP measurement using the Schioetz Tonometer in 22 patients [11], the correlation between ICP and IOP was only found in 2 patients. No significant correlation between the average IOP for both eyes and ICP was also well demonstrated in an another study [12].

## Conclusion

In summary, although a significant correlation exists between ICP and IOP, caution needs to be taken when using a single IOP measurement with Goldmann applanation tonometer to be an surrogate for direct CSFP measurements of ICP.

## Misc

Zhen Li, Yingxin Yang contributed equally to this work.

## Competing interests

The authors declare that they have no competing interests regarding study in question.

## Authors’ contributions

ZL, YY, DY, JJ and NW made substantial contribution to the study design, data collection, manuscript preparation and written up. ZL,YY and DY also contributed to the data collection. ZL, HY YY and DY completed with all the ophthalmic examinations while YL, DL and DM were involved in data analysis and interpretation of data of ophthalmic examinations. While EX and XZ were involved in analysis and interpretation of data of neurological examination, DM gave critical comments and a help for the manuscript written up. All authors gave approval of the final version to be published.

## Pre-publication history

The pre-publication history for this paper can be accessed here:

http://www.biomedcentral.com/1471-2377/12/66/prepub

## References

[B1] Pickard JD Czosnyka M Management of raised intracranial pressure J Neurol Neurosurg Psychiatry 1993 56 8 845 858 10.1136/jnnp.56.8.845 8350099 PMC1015137

[B2] Bellner J Romner B Reinstrup P Kristiansson KA Ryding E Brandt L Transcranial Doppler sonography pulsatility index (PI) reflects intracranial pressure (ICP) Surg Neurol 2004 62 1 45 51 51 10.1016/j.surneu.2003.12.007 15226070

[B3] Gumerlock MK York D Durkis D Visual evoked responses as a monitor of intracranial pressure during hyperosmolar blood–brain barrier disruption Acta Neurochir Suppl (Wien) 1994 60 132 135 7976525 10.1007/978-3-7091-9334-1_35

[B4] Foltz EL Blanks JP McPherson DL Hydrocephalus: increased intracranial pressure and brain stem auditory evoked responses in the hydrocephalic rabbit Neurosurgery 1987 20 2 211 218 10.1227/00006123-198702000-00002 3561726

[B5] Querfurth HW Lieberman P Arms S Mundell S Bennett M van Horne C Ophthalmodynamometry for ICP prediction and pilot test on Mt Everest. BMC Neurol 2010 10 106 10.1186/1471-2377-10-106 PMC2987855 21040572

[B6] Kast R A new method for noninvasive measurement of short-term cerebrospinal fluid pressure changes in humans J Neurol 1985 232 4 260 261 10.1007/BF00313790 4045519

[B7] Lashutka MK Chandra A Murray HN Phillips GS Hiestand BC The relationship of intraocular pressure to intracranial pressure Ann Emerg Med 2004 43 5 585 591 10.1016/j.annemergmed.2003.12.006 15111918

[B8] Sajjadi SA Harirchian MH Sheikhbahaei N Mohebbi MR Malekmadani MH Saberi H The relation between intracranial and intraocular pressures: study of 50 patients Ann Neurol 2006 59 5 867 870 10.1002/ana.20856 16634008

[B9] Spentzas T Henricksen J Patters AB Chaum E Correlation of intraocular pressure with intracranial pressure in children with severe head injuries Pediatr Crit Care Med 2010 11 5 593 598 10.1097/PCC.0b013e3181ce755c 20081553

[B10] Han Y McCulley TJ Horton JC No correlation between intraocular pressure and intracranial pressure Ann Neurol 2008 64 2 221 224 10.1002/ana.21416 18570302

[B11] Czarnik T Gawda R Latka D Kolodziej W Sznajd-Weron K Weron R Noninvasive measurement of intracranial pressure: is it possible? J Trauma 2007 62 1 207 211 10.1097/01.ta.0000219128.29515.d5 17215756

[B12] Kirk T Jones K Miller S Corbett J Measurement of intraocular and intracranial pressure: is there a relationship? Ann Neurol 2011 70 2 323 326 10.1002/ana.22414 21710618

[B13] Ren R Zhang X Wang N Li B Tian G Jonas JB Cerebrospinal fluid pressure in ocular hypertension Acta Ophthalmol 2011 89 2 e142 e148 10.1111/j.1755-3768.2010.02015.x 21348961

[B14] Sheeran P Bland JM Hall GM Intraocular pressure changes and alterations in intracranial pressure Lancet 2000 355 9207 899 10.1016/S0140-6736(99)02768-3 10752710

[B15] Lehman RA Krupin T Podos SM Experimental effect of intracranial hypertension upon intraocular pressure J Neurosurg 1972 36 1 60 66 10.3171/jns.1972.36.1.0060 4621385

[B16] Smith RB Aass AA Nemoto EM Intraocular and intracranial pressure during respiratory alkalosis and acidosis Br J Anaesth 1981 53 9 967 972 10.1093/bja/53.9.967 6793054

[B17] Nakano J Chang AC Fisher RG Effects of prostaglandins E 1, E 2, A 1, A 2, and F 2 on canine carotid arterial blood flow, cerebrospinal fluid pressure, and intraocular pressure J Neurosurg 1973 38 1 32 39 10.3171/jns.1973.38.1.0032 4682351

[B18] Salman MS Can intracranial pressure be measured non-invasively? Lancet 1997 350 9088 1367 9365458 10.1016/S0140-6736(05)65138-0

[B19] Madill SA Connor SE Computed tomography demonstrates short axial globe length in cases with idiopathic intracranial hypertension J Neuroophthalmol 2005 25 3 180 184 10.1097/01.wno.0000177302.90586.16 16148623

[B20] Brodsky MC Vaphiades M Magnetic resonance imaging in pseudotumor cerebri Ophthalmology 1998 105 9 1686 1693 10.1016/S0161-6420(98)99039-X 9754178

[B21] Dickerman RD Smith GH Langham-Roof L McConathy WJ East JW Smith AB Intra-ocular pressure changes during maximal isometric contraction: does this reflect intra-cranial pressure or retinal venous pressure? Neurol Res 1999 21 3 243 246 10319330 10.1080/01616412.1999.11740925

